# Transcription-coupled recruitment of human CHD1 and CHD2 influences chromatin accessibility and histone H3 and H3.3 occupancy at active chromatin regions

**DOI:** 10.1186/1756-8935-8-4

**Published:** 2015-01-15

**Authors:** Lee Siggens, Lina Cordeddu, Michelle Rönnerblad, Andreas Lennartsson, Karl Ekwall

**Affiliations:** Department of Biosciences and Nutrition, NOVUM, Karolinska Institutet, Huddinge, 141 83 Sweden

**Keywords:** chromatin remodeling, CHD1, CHD2, ENCODE, DNase, H3, H3.3

## Abstract

**Background:**

CHD1 and CHD2 chromatin remodeling enzymes play important roles in development, cancer and differentiation. At a molecular level, the mechanisms are not fully understood but include transcriptional regulation, nucleosome organization and turnover.

**Results:**

Here we show human CHD1 and CHD2 enzymes co-occupy active chromatin regions associated with transcription start sites (TSS), enhancer like regions and active tRNA genes. We demonstrate that their recruitment is transcription-coupled. CHD1 and CHD2 show distinct binding profiles across active TSS regions. Depletion of CHD1 influences chromatin accessibility at TSS and enhancer-like chromatin regions. CHD2 depletion causes increased histone H3 and reduced histone variant H3.3 occupancy.

**Conclusions:**

We conclude that transcription-coupled recruitment of CHD1 and CHD2 occurs at transcribed gene TSSs and at intragenic and intergenic enhancer-like sites. The recruitment of CHD1 and CHD2 regulates the architecture of active chromatin regions through chromatin accessibility and nucleosome disassembly.

**Electronic supplementary material:**

The online version of this article (doi:10.1186/1756-8935-8-4) contains supplementary material, which is available to authorized users.

## Background

ATP-dependent chromatin remodeling enzymes disrupt DNA-histone interactions facilitating nucleosome mobilization, including the disassembly, eviction, sliding and spacing of nucleosomes [1]. The ability of ATP-dependent chromatin remodeling enzymes to manipulate nucleosomes also facilitates diverse chromatin-associated processes including transcription, DNA repair, incorporation of histone variants, DNA methylation, the covalent modification of histone tails and gene activation or silencing [2]. All ATP-dependent chromatin remodeling enzymes contain a SNF2-like helicase domain [3]. The chromodomain helicase DNA binding protein (CHD) subfamily of ATP-dependent chromatin remodeling enzymes are characterized by the presence of dual chromatin organization modifier (chromo) domains N-terminal to the SNF2 domain [4]. Human CHD1 and CHD2 are grouped as subclass I CHD remodelers based on both their modular organization, with N-terminal dual chromodomains and on their sequence similarity within the SNF2 helicase domain in relation to CHD1 homologues in other organisms [3, 5]. In contrast, other human CHD family remodelers are grouped into class II and class III CHD proteins on the basis of additional regulatory domains such as plant homeobox domains as well as protein sequence differences in the SNF2 helicase domain [3, 5].

Mammalian CHD1 has important biological roles in stem cell function, prostate cancer and transcription, while CHD2 has emerged as an important regulator of genetic stability, development, kidney function, muscle cell and hematopoietic differentiation [6–15]. In model organisms, studies on CHD1 homologues have demonstrated various mechanistic roles in nucleosome disassembly, histone turnover, nucleosome spacing, transcription through chromatin, transcription-coupled chromatin assembly and deposition of the histone H3 variant H3.3 during development [10, 16–19]. Relatively little is known about CHD2 function on a molecular level with the exception of muscle cell differentiation where it was demonstrated that CHD2 is an essential cofactor for the transcription factor MyoD in H3.3 deposition at muscle cell gene promoters [12].

Interestingly, CHD family remodelers function as chromatin readers in some cases by recognizing specific histone modifications [4]. For example human CHD1 has specific affinity for histone 3 (H3) tails when di- or tri-methylated at lysine 4 (H3K4me2 and H3K4me3) [20]. The role of H3K4me2/3 in the physical recruitment of CHD1 *per se* is not clear although both are present at transcribed gene promoters [4]. For example, some studies have demonstrated that CHD1 recruitment is at least partially independent of the chromodomains [21, 22], while other studies have linked CHD1 to chromatin associated with transcriptional elongation [23–25]. In the model organisms, budding yeast and Drosophila, CHD1 homologues do not show any specific affinity for H3K4me2/3 and bind unmodified H3 [20, 21].

The fission yeast CHD1 homologue Hrp1 was shown to co-purify with the fission yeast mediator complex [26]. Consistent with this finding, it was later demonstrated that mediator-dependent assembly of the pre-initiation complex *in vitro* is associated with CHD1 recruitment in mammalian cells [9]. Surprisingly, the latter study demonstrated CHD1 recruitment to pre-initiation complexes on both naked DNA and chromatin-based templates, including chromatin templates with and without H3K4me3 [9]. Unlike CHD1, the chromodomains of CHD2 do not have specific affinity H3K4me3 [27]. In mouse and human cells, the myogenic transcription factor MyoD is sufficient for the recruitment of CHD2 to muscle lineage gene promoters, which may be mediated through a direct interaction between MyoD and CHD2 [12]. It was also suggested that CHD2 does not bind to housekeeping genes, but operates as a muscle specific factor in muscle lineage activation. Thus, exactly how chromatin remodeling enzymes such as CHD1 and CHD2 are recruited to target sites remains an open question.

To examine how subclass I CHD remodelers, CHD1 and CHD2 are recruited and function in human cells, we first carefully examined the occupancy of each in relation to chromatin states utilizing data from the Encyclopedia of DNA Elements (ENCODE) consortium (http://genome.ucsc.edu/ENCODE/). Closer inspection of the data demonstrates that neither H3K4me3 nor micrococcal nuclease (MNase) sensitive DNA at the NDR is correlated to CHD1 or CHD2. We demonstrate that both CHD1 and CHD2 are recruited in association with the RNA polymerase II (Pol II) machinery to active chromatin regions. Depletion of CHD1 in human cells reduces the accessibility of active regions as measured by DNase sensitivity. We also show that at active chromatin regions H3 occupancy is increased following knockdown of CHD2 suggesting a role in promoting nucleosome disassembly at active regions genome-wide. Knockdown of CHD2 also leads to a reduction in the relative enrichment of H3.3 further supporting a role for CHD2 in nucleosome turnover. We speculate that noncoding transcription at regulatory elements such as promoters, enhancers and active tRNA loci functions partially to modulate the chromatin environment through the recruitment and action of transcription coupled factors such as the remodeling enzymes CHD1 and CHD2.

## Results and discussion

### Recruitment of CHD1 and CHD2 to promoters is linked to the Pol II machinery

**Figure 1:**
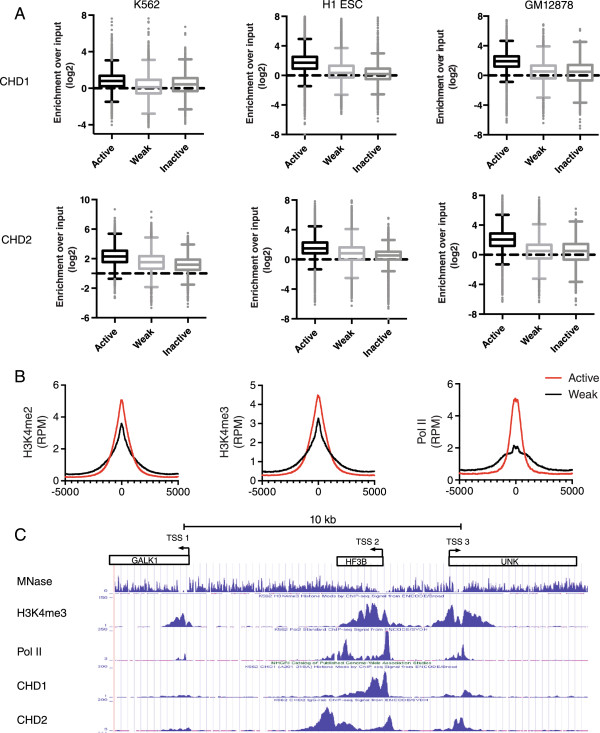
**CHD1 and CHD2 are recruited to active but not weak or inactive promoters in association with Pol II. (A)** CHD1 and CHD2 occupancy is strongest in the active promoter chromatin state in K562 H1 embryonic stem cell (ESC) and GM12878 cells. CHD1 and CHD2 chromatin immunoprecipitation high throughput sequencing (ChIP-seq) data were normalized to input. Box plots illustrate the median and 25th to 75th percentile with whisker length determined by the Tukey method. In all cell types examined for both CHD1 and CHD2, enrichment at active promoters is statistically higher than at weak and inactive promoters (Kruskall-Wallis test with Dunn’s post test correction, k = 3, *P* <0.0001) **(B)** H3K4me2 and H3K4me3 are present at both active and weak promoters, while Pol II enrichment distinguishes the active and weak promoter chromatin state. **(C)** Examples of the transcription start site (TSS) of genes marked by H3K4me2/3 with differential Pol II, CHD1 and CHD2 binding. The GAL1K1 promoter - TSS1 is marked by H3K4me2/3 but is not bound by Pol II, CHD1 or CHD2, while the H3F3B promoter, TSS2, is strongly bound by Pol II along with CHD1 and CHD2. TSS3 of the UNK gene promoter displays H3K4me2/3 enrichment on a similar scale to the H3F3B gene but has weaker Pol II, CHD1 and CHD2 binding.

We analyzed CHD1 and CHD2 occupancy by calculating the enrichment of CHD1 and CHD2 from ChIP-seq data in relation to the chromatin states delineated by Ernst *et al.* [28]. The chromatin states established by Ernst *et al.* were based on ChIP-seq data of combinatorial patterns of eight histone modifications and the insulator binding protein CTCF [28]. The promoter and enhancer chromatin states are summarized in Table 1. In relation to promoter chromatin states; H3K4me2/3 and high H3K9/K27ac define the active promoter state. The weak promoter state is lower in H3K9/27 ac compared to the active promoter state, and H3K27me3 defines the inactive promoter. Enrichment of CHD1 and CHD2 was strongest at the active promoter chromatin state (Figure 1A). In all three cell types examined for both CHD1 and CHD2, the enrichment observed at active promoters was significantly higher than at weak or inactive promoters (Kruskall-Wallis, k = 3, *P* <0.0001). Previous studies have demonstrated in model organisms and human cells that CHD1 remodelers are recruited to promoters in a gene expression-dependent manner [29, 30]. Importantly, the weak promoter state from the ENCODE project is also defined by H3K4me2/me3 [28]. This suggested that H3K4 methylation is therefore not sufficient for recruiting CHD1 to promoters *in vivo* since occupancy is strongly reduced or absent at weak promoters, which are marked by H3K4me2/3.

**Table 1:** **Summary of promoter and enhancer chromatin states identified by Ernst** ***et al.*** **in relation to histone methylation and acetylation**

Chromatin state	H3K4me1	H3K4me2	H3K4me3	H3K27me3	H3K9ac	H3K27ac
**Active promoter**	**+**	**+++**	**+++**	**-**	**+++**	**+++**
**Weak Promoter**	**++**	**+++**	**+++**	**-**	**+**	**++**
**Inactive promoter**	**++**	**+++**	**++**	**+++**	**-**	**+**
**Enhancer state 4**	**+++**	**+++**	**++**	**-**	**+++**	**+++**
**Enhancer state 5**	**+++**	**++**	**-**	**-**	**+**	**+++**
**Enhancer state 6**	**++**	**+++**	**-**	**-**	**-**	**-**
**Enhancer state 7**	**++**	**-**	**-**	**-**	**-**	**-**

The frequency of a given mark at each chromatin state is identified as not present (-; <5% frequency), low (+; 5 to 40% frequency), abundant (++; 40 to 70% frequency) or highly abundant (+++; 70 to 100% frequency).

We hypothesized that the presence and absence of the Pol II transcription machinery occupancy may explain the difference in occupancy observed between the active and weak promoter states respectively. Consistent with this notion, Pol II was strongly enriched at active promoters only, whereas the H3K4me2/3 levels were comparable at both promoter states (Figure 1B). This was also apparent when inspecting individual gene promoters. In K562 cells at the GALK1 transcription start site (TSS), (Figure 1C; TSS 1), MNase-sequencing data and ChIP-seq data showed low nucleosome occupancy and high levels of H3K4me2/3, yet Pol II, CHD1 and CHD2 were not bound. In contrast, the TSS and gene body of the highly transcribed H3F3B (Figure 1C; TSS 2) gene were bound by Pol II and strongly enriched for the CHD1 and CHD2 remodelers. The UNK gene TSS (Figure 1C; TSS 3) showed lower levels of Pol II, CHD1 and CHD2 occupancy but abundant H3K4me2/3. Such examples clearly demonstrated that Pol II levels associate with CHD1 and CHD2 occupancy, indicating that transcription-coupled recruitment of CHD1 and CHD2 is important in targeting the enzymes to active promoters. Morettini *et al.* demonstrated that the chromodomains of *Drosophila melanogaster* CHD1 are critical for CHD1 function but not recruitment and concluded that ChIP-seq studies were needed to confirm this [21]. Our analysis of human CHD1 provides support for this conclusion. Studies on CHD7, which is also reported to bind H3K4me2/3, revealed that CHD7 occupancy occurs on a fraction of H3K4me2/3 sites [31, 32]. These studies and the CHD7 data available in the ENCODE project suggest that CHD7, like CHD1, is recruited to a fraction of H3K4me2/3 sites independently of the chromodomain-H3K4me2/3 interaction since at sites such as weak promoters marked by H3K4me3, CHD7 is not recruited (see Additional file 1).

**Figure 2:**
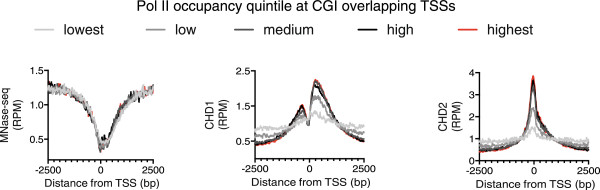
**Recruitment of CHD1 and CHD2 at CpG island (CGI) promoters correlates with RNA polymerase II (Pol II) occupancy independently of micrococcal nuclease (MNase) sensitivity.** Genes with CGI overlapping the transcription start sites (TSS) were organized into quintiles of ascending Pol II occupancy in K562 cells. The average MNase-seq signal, CHD1 and CHD2 occupancy was then plotted across the region from -2500 to +2500 bases from the annotated TSS. Light gray lines represent the bottom quintile, medium gray the second lowest quintile, dark gray the third quintile, black the fourth and red the fifth quintile of Pol II occupancy.

CHD remodeling enzymes also contain nonsequence-specific DNA binding domains, which play important roles in the regulation of chromatin remodeling. Zentner *et al.* hypothesized that this may drive recruitment of the CHD family of remodeling enzymes to active promoters, where nucleosome disassembly provides naked DNA [30]. In human cells, the majority of promoters have CpG islands (CGI), which consist of stretches of abundant CpG base pairs. Such promoters are MNase sensitive and are thought to have constitutively lower nucleosome occupancies relative to non-CGI promoters independently of transcription rates [33]. Thus, in mammalian cells, nucleosome occupancy and transcription can be partially uncoupled since non-CGI promoters show low nucleosome occupancy only when highly transcribed, while CGI promoters are constitutively MNase sensitive [33]. Grouping of CGI promoters into quintiles of ascending Pol II occupancy clearly demonstrated that CHD1 and CHD2 recruitment is correlated to Pol II occupancy at these sites (Figure 2). Furthermore, despite showing low nucleosome occupancy in the absence of transcription, CGI TSSs do not recruit CHD1 or CHD2; that is, the accessible DNA at these nucleosome-depleted regions (NDRs) is not sufficient for the recruitment.

### Recruitment of CHD1 and CHD2 at enhancer-like chromatin and active tRNA genes

**Figure 3:**
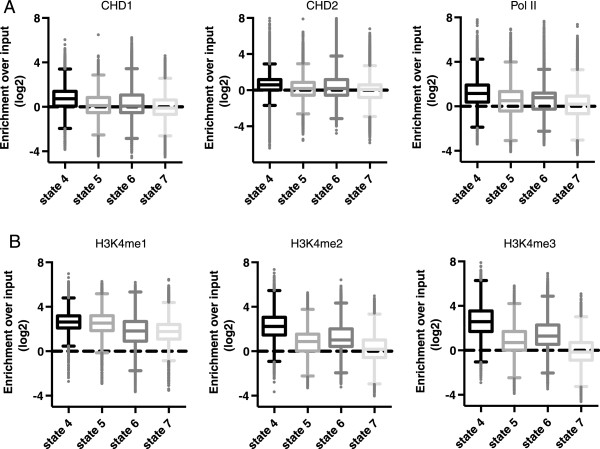
**CHD1 and CHD2 are enriched at an enhancer-like chromatin state with RNA polymerase II (Pol II). (A)**The occupancy of CHD1 and CHD2 occurs primarily at enhancer-like chromatin state 4, which also showed the strongest binding by Pol II (Kruskall-Wallis test with Dunn’s post test correction, k = 3, *P* <0.0001, comparing enhancer state 4 to all other enhancer-like chromatin states). **(B)** In contrast H3K4 methylation was observed to different extents at all enhancer-like chromatin states.

Another chromatin state strongly associated with CHD1 or CHD2 enrichment was enhancer chromatin state 4 (Figure 3A). The enhancer chromatin states defined by Ernst *et al.* were identified through combinations of H3K4-methylation including the enhancer-specific H3K4me1 and histone acetylation (Table 1, Figure 3B). We found that enhancer state 4, which is distinguished by higher H3K4me3 and H3K9/27 ac than other enhancer states, was associated with Pol II, CHD1 and CHD2 enrichment. Other enhancer chromatin states 5 to 7, defined by lower levels of H3K4 methylation and H3K9/27 ac, showed weaker enrichment for Pol II, CHD1 and CHD2. This suggested that transcription of enhancers is coupled to chromatin remodeling by CHD1 and CHD2. Furthermore it has been demonstrated that a fraction of Pol II binding sites overlap with a distinct set of RNA polymerase III binding sites including active tRNA genes [34, 35]. Such active tRNA genes are bound by BRF1, the transcription factor IIIB subunit [36]. Consistent with this, we found CHD1 and CHD2 were enriched at BRF1 but not at the alternate Pol III transcription factor, BRF2 binding sites in K562 cells (see Additional file 2). In this scenario, BRF2 binding sites function as control Pol III sites that do not overlap with Pol II binding. This suggests CHD1 and CHD2 may function at a subset of Pol III genes where Pol II transcription occurs, such as tRNAs.

### CHD1 and CHD2 show overlapping occupancy but discrete binding profiles at transcription start sites

**Figure 4:**
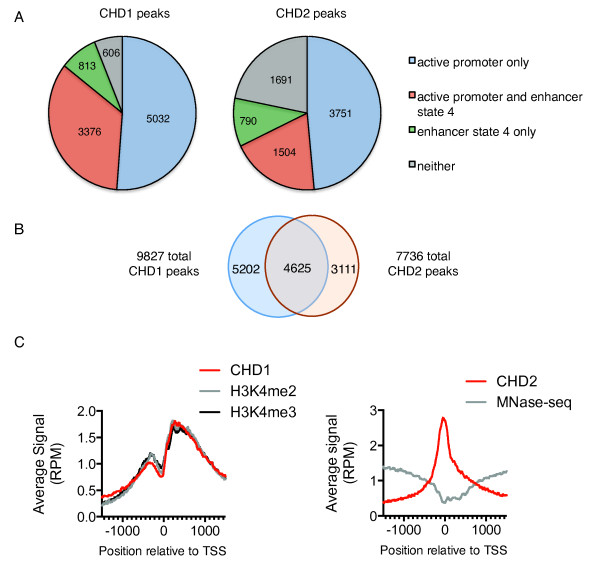
**CHD1 and CHD2 co-occupy active promoters but show different binding profiles across the transcription start site (TSS). (A)** Statistically significant high confidence peaks from CHD1 and CHD2 chromatin immunoprecipitation sequencing (ChIP-seq) data were intersected with chromatin state annotations from K562 cells using model-based analysis for ChIP-seq (MACS) with a stringent cut-off p value of 1 x 10^-15^. The majority of CHD1 (94%) and CHD2 (78%) binding sites fall within an active promoter or enhancer chromatin state or both as defined by Ernst *et al.* **(B)** The majority of CHD1 and CHD2 bound sites are overlapping; 60% of the 7,736 CHD2 binding sites overlap with at least one of 9,827 CHD1 peaks. **(C)** At highly expressed genes defined in the top 20% expression quintile, CHD1 binding profile follows the enrichment of H3k4me2/3-marked nucleosomes. In contrast, CHD2 binds strongest across the micrococcal nuclease (MNase) sensitive nucleosome depleted region (NDR) at the TSS.

To validate the finding that CHD1 and CHD2 both prefer active promoter and enhancer chromatin states we identified ChIP-seq peaks using model-based analysis for ChIP-seq (MACS) [37] with a stringent cut-off p value of 1 × 10^-15^. For both data sets, peaks were merged if they fell within approximately one nucleosome length from another (200 bp). This produced 9,827 CHD1 peaks and 7,736 CHD2 peaks. For CHD1 and CHD2 respectively, 94% and 78% of high confidence peaks fell within a region associated with the active promoter or enhancer chromatin state 4 or both, based on chromatin states defined by Ernst *et al.* (Figure 4A, Table 1). Since chromatin states are continuous, a given peak may fall across more than one chromatin state.

The majority of CHD1 and CHD2 sites overlapped (60%) (Figure 4B). Despite this, the exact binding profiles of CHD1 and CHD2 at transcription start sites of genes in the top expression quintile differed (Figure 4C). CHD1 was enriched in a manner mimicking its preferred H3K4me2/3 nucleosomal substrates, while CHD2 bound across the NDR (Figure 4C). For CHD1, we propose that transcription-coupled recruitment with the Pol II machinery at the pre-initiation complex stage functions as the primary recruitment mechanism. In support of this, it is well established that CHD1 interacts with multiple Pol II-associated complexes [9, 22, 23, 38, 39]. Following recruitment, CHD1 then presumably interacts preferentially with H3K4me2/3 marked nucleosomes. In contrast, CHD2 does not have any specific affinity for H3K4me2/3 and binds strongest at the NDR. An alternative possibility is that differences in cross-linking preferences could affect the results obtained from conventional ChIP-seq. Future studies could address this by using higher resolution techniques, such as those employed by Skene *et al.*, for murine Chd1 or using ChIP-exo to gain a more exact chromatin remodeler-DNA footprint [29, 40].

**Figure 5:**
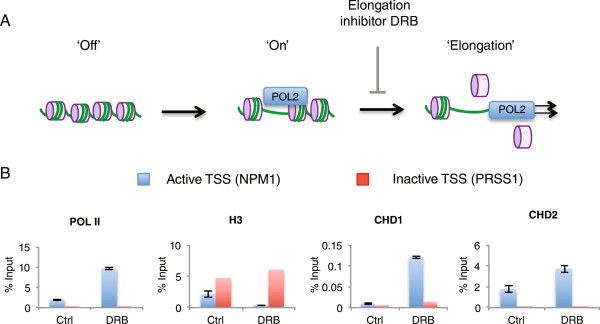
**Prolonged RNA polymerase II (Pol II) elongation inhibition leads to an accumulation of Pol II, a reduction in nucleosome occupancy and recruitment of CHD1 and CHD2. (A)** Chromatin immunoprecipitation sequencing (ChIP-seq) data in asynchronous cultures reflect the complex dynamics associated with cells at different stages of transcription such as the inactive off state, the initiation stage when the Pol II machinery assembles and the elongation phase following promoter escape. Elongation inhibitor 5,6-dichloro-1-β-D-ribofuranosyl-1H-benzimidazole (DRB) synchronizes transcription in cells by preventing elongation and promoter escape. **(B)** Prolonged DRB inhibition results in Pol II accumulation and H3 depletion at the transcription start site (TSS) of an expressed gene (NPM1) but not inactive gene (PRSS1). Pol II accumulation associates with increased CHD1 and CHD2 recruitment (significant differences between means of control and DRB tested with the students t-test, ***P <0.001).

To test if transcription was linked to CHD1 and CHD2 recruitment, K562 cells were incubated with the Pol II CTD kinase inhibitor 5,6-dichloro-1-β-D-ribofuranosyl-1H-benzimidazole (DRB) to block elongation and synchronize transcription at the initiation stage (Figure 5A). This led to an accumulation of Pol II and a reduced H3 occupancy at the active NPM1 gene promoter as measured by ChIP-qPCR (Figure 5B, Students t-test, ***P <0.001). The inactive PRSS1 gene promoter was used as a control. Consistent with the proposed model of Pol II-driven recruitment, the binding of both CHD1 and CHD2 to the NPM1 gene promoter was significantly increased (Figure 5B, Students t-test ***P <0.001). Thus, we conclude that CHD1 and CHD2 are recruited with the Pol II machinery. The Henikoff laboratory recently demonstrated that a catalytically inactive, ATPase mutant of murine Chd1 reduces Pol II release from promoters, resulting in increased Pol II occupancy at TSSs. Consistent with our data (Figure 5B) increased Pol II occupancy in cells expressing mutant Chd1 was associated with increased Chd1 occupancy directly across the TSS [29]. Our findings are also consistent with a recently published study showing that budding yeast Chd1 more closely matches Pol II phosphorylated at Serine 5 across the 5’ regions of genes than it does with the elongating form phosphorylated at Serine 2 [41]. This is the case for both human CHD1 and CHD2, which bind across the 5’ region of genes similar to total Pol II ChIP-seq data while serine 2 phosphorylated Pol II shows stronger enrichment at the 3’ end of genes (see Additional file 3).

### CHD1 affects chromatin accessibility at transcription start, intragenic and intergenic sites

To begin to dissect the functions of CHD1 and CHD2 and to confirm that they target active loci such as promoters and enhancers, we performed siRNA knockdowns in K562 cells combined with DNase accessibility assays. Effective knockdown of CHD1 and CHD2 was demonstrated at both mRNA and protein levels (Additional file 4).

**Figure 6:**
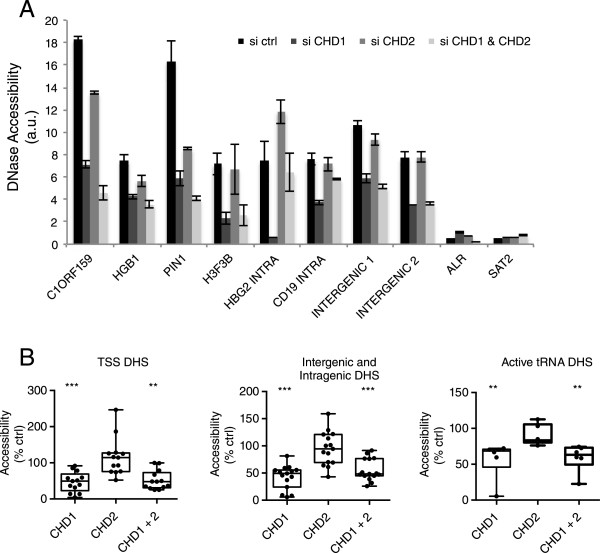
**CHD1 regulates chromatin accessibility at transcription start site (TSS)-associated, intergenic, intragenic and active tRNA DNase hypersensitivity sites (DHS). (A-B)** Knockdown of CHD1 reduces the accessibility of TSS-associated, intragenic and intergenic DHS sites in K562 cells. **(A)** Data showing the accessibility of selected loci representing non-CGI TSS DHS sites (C1ORF159 and HGB1) CGI TSS sites (PIN1 and H3F3B), intragenic DHS sites (HGB2 and CD19 intra) and two intergenic loci. Centromeric repeats were used as inaccessible controls. Data represent biological replicates and error bars standard deviation. **(B)** Mean accessibility values from three biological replicates for each loci tested were plotted as the percentage of accessibility in control siRNA treated cells. Differences compared to control were tested by one-way ANOVA, **P <0.01, ***P <0.001.

DNase hypersensitivity sites (DHS) define regions of accessible chromatin associated with TSSs of expressed genes, while intergenic and intragenic DHS represent potential enhancer regions associated with transcription factor binding sites [42]. We analyzed ENCODE DHS associated with the TSSs of highly expressed genes in K562 cells (n = 15) as well as intergenic and intragenic DHS sites (n = 15) and expressed tRNA genes (n = 6), which overlap DHS sites. Depletion of CHD1 with siRNA reduced the accessibility of all DHS types tested, while CHD2 knockdown did not (Figure 6A-B, **P <0.01, ***P >0.001, one-way ANOVA). Inaccessible centromeric repeats were used as a negative control and remained inaccessible (Figure 6A). The decrease in accessibility of DHS sites in CHD1 siRNA treated K562 cells was reduced to less than 10% to more than 90% of control (Figure 6B). Yet, since all DHS sites tested remained accessible to DNase to some degree, other factors must operate independently of CHD1 to promote accessible chromatin. Furthermore since there are on average more than 200,000 DHS loci per cell type [42], and we only tested 36 DHS sites, this study is not comprehensive. It does suggest, however, that CHD1 co-operates with other factors to promote accessible chromatin at regulatory regions.

DHS are embedded within chromatin marked by H3K4me2/3 at TSS and at intergenic and intragenic DHS sites including enhancers [43–46]. One possible function of CHD1 in light of the role in promoting chromatin accessibility could be to facilitate the binding of other transcription factors and complexes. This has been demonstrated in the case of post-initiation factors such as the splicing machinery, whereby CHD1 promotes efficient recruitment [47]. Interestingly accessible chromatin is associated with both nucleosome-free and nucleosome containing regions and one of the strongest correlations between chromatin accessibility and histone modifications is the positioning and strength of the H3K4me3 nucleosomes, which may act to promote CHD1-mediated mobilization or relaxation of nucleosomes at regulatory regions [48].

### CHD1 and CHD2 differentially affect H3 and H3.3 occupancy

One possible explanation for the effect of CHD1 on chromatin accessibility could be through the disassembly of nucleosomes. In fission yeast, the CHD1 homologues Hrp1 and Hrp3 function to reduce H3 occupancy at promoter regions [10, 19]. In addition, the H3 variant H3.3 may act to promote chromatin accessibility in some circumstances, and CHD1 homologues in model organisms and mammalian CHD2 have both been linked to H3.3 deposition [12, 16].

To analyze H3 occupancy and H3.3 occupancy by ChIP-qPCR, we first tested the specificity of the total H3 and H3.3 antibodies with recombinant human H3.1 and H3.3 proteins. The anti-H3 antibody detected both H3.1 and H3.3, while the H3.3 specific antibody detected only recombinant H3.3 protein by western blotting (see Additional file 5). We then tested the performance of these antibodies by ChIP-qPCR. When normalizing to total H3 occupancy, which is reduced at active regions compared to inactive regions, the relative enrichment of H3.3 was higher at the active GAPDH promoter compared to inactive MYOD promoter (see Additional file 5). Similarly, at the NPM1 gene, the H3.3/H3 total ratio was higher at the promoter compared to an intron from the gene body (see Additional file 4).

**Figure 7:**
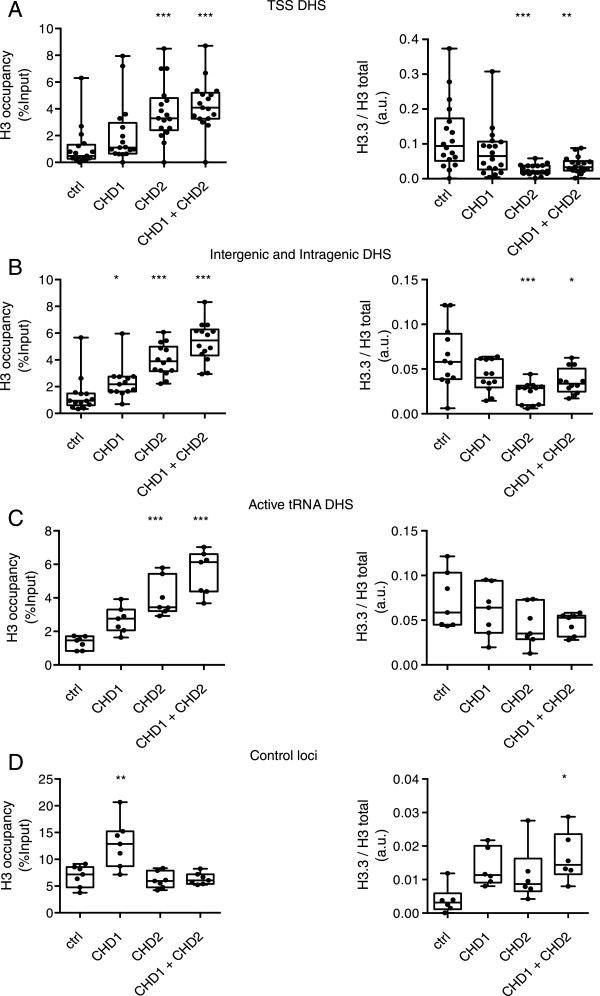
**CHD1 and CHD2 control H3 and H3.3 occupancy at active regulatory regions. (A)** Chromatin immunoprecipitation (ChIP-qPCR) analysis of control CHD1, CHD2 and double knockdown experiments at promoters associated with DNase hypersensitivity sites (DHS) show increased H3 occupancy with CHD1, CHD2 and double knockdowns in K562 cells. The strongest effect on H3 occupancy is observed with CHD2 knockdown. CHD2 knockdown also reduced H3.3 enrichment. **(B)** At intergenic and intragenic DNase sensitivity sites CHD1 and CHD2 knockdown increase H3 occupancy with the strongest trend observed in CHD2 and double knockdown cells. As for promoter sites, CHD2 knockdown reduced H3.3 enrichment. **(C)** Active tRNA loci-associated DNase sensitive sites show similar trends for both H3 and H3.3 occupancy. **(D)** Control loci from nonaccessible chromatin demonstrate the effects of CHD2 knockdown on H3 and H3.3 occupancy are specific to active regions. All data points represent mean values plotted from three biological replicates per loci tested. Differences compared to control were tested by one-way ANOVA, *P <0.05, **P <0.01, ***P <0.001.

We selected TSS, intragenic, intergenic and tRNA-associated DHS sites, as well as a number of non-DHS control loci. for analysis by ChIP-qPCR (Figure 7). At TSSs, CHD1 knockdown lead to a nonsignificant trend toward increased H3 occupancy, while CHD2 knockdown lead to a striking increase in total H3 occupancy (Figure 7A, ***P > 0.001, one-way ANOVA). Double CHD1 and CHD2 knockdowns showed an additive effect with an elevated H3 occupancy compared to the single CHD2 knockdown (Figure 7A, 3.8% input compared to 4.3% input). In contrast the relative H3.3 enrichment observed in CHD2 and double CHD1 and CHD2 knockdowns was significantly lower than that of control and CHD1 knockdowns (Figure 7A, ***P <0.001, **P <0.01, one-way ANOVA). Similar results were obtained at intragenic and intergenic DHS sites with CHD2 knockdown giving significantly elevated H3 occupancy and reduced H3.3 enrichment (Figure 7B, *P <0.05, ****P <0.0001, one-way ANOVA). Similar trends were also observed at active tRNA loci associated DHS for both H3 and H3.3 (H3 ***P <0.001, H3.3, n.s. one-way ANOVA).

Surprisingly, DNase accessibility, H3 occupancy and H3.3 enrichment do not appear to be directly related in this study since, CHD1 affects accessibility strongly with weaker affects on H3 occupancy, while CHD2 affects H3 occupancy and H3.3 enrichment without affecting accessibility. Nucleosome occupancy is not the only factor that regulates chromatin accessibility. Accessible chromatin sites at intergenic loci for example show higher nucleosome occupancy and smaller nucleosome-free regions than transcription start sites but are still highly accessible [48, 49]. Accessible regulatory regions are composed of both nucleosome-free transcription factor binding sites and adjacent accessible nucleosomes [48, 49]. Hormone-inducible transcription factors bind to nucleosome-containing, accessible sites and are associated with chromatin remodeling but not necessarily nucleosome eviction [43, 50]. For example, nucleosomes are present at accessible intergenic progesterone receptor binding sites; the ensuing progesterone receptor activation accessibility is increased while total H3 occupancy remains unaltered [50]. It is also possible that CHD1 depletion leads to abnormalities in higher order chromatin structure in a similar manner to what was demonstrated in Drosophila polytene chromosomes, following CHD1 deletion [51].

It is perhaps less surprising that the strong increase in H3 occupancy does not restrict chromatin accessibility since previous studies in fission yeast revealed that *hrp1* and *hrp3* deletions increase promoter H3 occupancy but do not affect MNase sensitivity of promoter regions [10, 19]. One possibility is that CHD2 reduces interactions of non-nucleosomal H3 with naked DNA. It also suggests that in the absence of CHD2, other factors prevent a mature and access restricting nucleosome from forming. Reduced H3.3 occupancy following CHD2 knockdown is likely a result of reduced CHD2-dependent nucleosome disassembly since it has been clearly demonstrated that H3.3 deposition correlates with histone turnover at active loci [52].

### Implications for the biological functions of CHD1 and CHD2

Our analysis of ChIP-seq data for the CHD1 and CHD2 in human cells helps to reinterpret previously suggested models of CHD1 and CHD2 recruitment. We show that the recruitment is transcription-coupled and not driven by H3K4me2/3 or binding to NDR regions. The primary mechanisms of CHD1 recruitment to promoters and active chromatin regions must be driven by interactions between CHD1 and the mediator and must facilitate chromatin transcription (FACT); polymerase associated factor (PAF); and Spt-, Ada-, and Gcn5 acetyltransferase (SAGA) complexes [22, 38, 39]. The chromodomains of CHD1 instead act in an important post-recruitment step in the activation and regulation of remodeling. Consistent with this notion, chromodomain-nucleosome interactions of CHD1 regulate DNA-dependent activation of the ATPase motor [53].

An important role of CHD1 is to facilitate Pol II transcription through chromatin, regulating nucleosome turnover and transcription coupled chromatin assembly [9, 18, 29]. The affinity of human CHD1 for H3K4me2/3 may target the remodeling function of CHD1 to the 5’ region of active genes. For example it was recently suggested that mammalian CHD1 plays a key role in promoting Pol II escapes from active promoters by alleviating the barrier of the +1 nucleosome [29].

CHD2 has been linked to genetic stability, DNA repair and differentiation. In muscle cell differentiation, the myogenic transcription factor MyoD requires CHD2 for H3.3 deposition into muscle specific gene promoters. The strongest effects were likely seen at muscle-specific genes in this study since these regions are heavily transcribed in the model used [12]. We observe that the H3.3 enrichment is not specific to muscle cell differentiation and speculate that CHD2 is an essential enzyme for the disassembly of H3 nucleosomes, which is a pre-requisite for the deposition of new H3.3 during differentiation. In other cell types it is possible that other transcription factors promote the recruitment of CHD2 in an analogous manner to MyoD. The effect of CHD1 and CHD2 in particular, on H3.3 occupancy suggest they may also play a role in the reassembly of H3.3 nucleosomes following transcription.

The transcription coupled recruitment of both CHD1 and CHD2 suggest that transcription could also play a role in noncoding regions of the genome such as promoters, enhancers, and active tRNA loci. Previous reports have suggested such functions for Pol II transcription at tRNA genes, leading to the hypothesis that Pol III transcription is facilitated by the Pol II associated machinery [34, 35]. Active enhancers are also identified by Pol II transcription [54]. In such instances, we speculate transcription may be a mechanism for modulating chromatin structure. It was demonstrated that another transcription-coupled, SNF2 chromatin-remodeling enzyme Brg1 (SMARCA4), was required for enhancer activation of the MYC gene in leukemia [55]. Another key question is how multiple remodeling factors determine the activity of each particular locus. A recent study examining distinct remodeling enzyme families demonstrated overlapping binding of multiple remodelers to active regions [56]. Despite redundancy at the level of genome-wide occupancy, inactive mutants of the murine chromatin remodelers Brg1, Chd4 and hSNF2 all regulated chromatin accessibility at a subset of loci [56].

## Conclusions

We conclude that CHD1 and CHD2 are recruited along with the transcription machinery to promoters and enhancer sites associated with transcription. For CHD1, this establishes a clear model of recruitment via interactions with Pol II-associated complexes. This provides a basis for interpretation of previous studies and models of CHD1 recruitment. At active regions, CHD1 and CHD2 cooperate to regulate the chromatin architecture. Thus, transcription coupled chromatin remodeling promotes chromatin accessibility and nucleosome disassembly at transcribed regulatory regions.

## Methods

### Analysis of ENCODE data

Data from the ENCODE project were accessed and downloaded through the UCSC web portal (http://genome.ucsc.edu/ENCODE/). The files used in this study are listed in Additional file 6. ChIP-seq and chromatin state data were analyzed using Seqmonk (http://www.bioinformatics.babraham.ac.uk/projects/seqmonk/). All ChIP-seq data, including CHD1 and CHD2 occupancy, was measured as the fold enrichment over input. Where indicated in the text, differences between chromatin states were tested for significance using the Kruskall-Wallis test with Dunn’s post-test correction. High confidence CHD1 and CHD2 peaks were calculated using model-based analysis for ChIP-seq (MACS) with a stringency cut off of 1 × 10^-15^ and binding sites within 200 bp or less were merged [37]. MACS was used from Seqmonk version 0.27.0. For probe trend plots calculating the enrichment of H3k4me2/3 and Pol II over active and weak promoters and CHD1 or CHD2 occupancy across TSS regions, read counts were normalized to the total number of reads and the presented as the average signal per probe.

### Cell culture and drug treatment

K562 cells were cultured in RPMI 1640 medium containing glutamine (Life Technologies, Stockholm, Sweden), which was supplemented with 10% fetal bovine serum, 10 U/mL penicillin and 10 U/mL streptomycin (Life Technologies). To arrest transcriptional elongation K562 cells were treated overnight (12 h) with 100 μM 5,6-dichloro-1-β-D-ribofuranosyl-1H-benzimidazole (DRB) (Sigma-Aldrich, Stockholm, Sweden) or vehicle (DMSO).

### siRNA knockdown and validation

siRNA targeting CHD1 (s2974 and s2975) and CHD2 (s2979), as well as nontargeting negative controls, were obtained from Life Technologies (Stockholm, Sweden. K562 cells were transfected with the Neon electroporation system (Life Technologies, Stockholm, Sweden; 100 ul kit). The transfection protocol was performed as per manufacturer’s instructions with the following electroporation parameters: three pulses, 1,450 v, and 10 ms pulse width. The final total concentration of siRNA per sample was 25 nM in 8 mL of antibiotic-free RPMI 1640 medium with 10% serum per 3 × 10^6^ cells.

### RT-qPCR

RNA from 5 × 10^6^ K562 cells was isolated using the RNeasy plus kit (QIAGEN, Sollentuna, Sweden). A total of 1 μg of RNA was reverse-transcribed using Superscript™ III First Strand Synthesis Supermix (Life Technologies, Stockholm, Sweden) according to the manufacturer’s instructions. Resultant cDNA was diluted 1:20, and qPCR analysis of CHD1 and CHD2 transcript abundance was normalized to GAPDH using Taqman assays (Life Technologies, Stockholm, Sweden; CHD1 assay ID - Hs00154405_m1; CHD2 assay ID - Hs00172280_m1; GAPDH assay ID - Hs02758991_g1).

### Western blotting

For western blotting, 5 × 10^6^ K562 were lyzed using single detergent lysis buffer (50 mM Tris, 150 mM NaCl, and 1% Triton X-100, pH 8.0) and protein concentration was calculated using the BCA protein assay (Fisher Scientific, Stockholm, Sweden). A total of 40 μg of protein per sample was denatured and resolved on a 4 to 12% Bis-Tris gel with MOPS running buffer (Novex, NuPAGE, precast gels, Life Technologies, Stockholm, Sweden). Gels were then wet-transferred to a 0.2-μM PVDF membrane (Millipore, Solna, Sweden) at a constant voltage of 100 V for 120 min. Membranes were air-dried, reactivated for 5 min in methanol and then blocked for 4 h at room temperature in 5% milk in PBS with 0.1% Tween. Membranes were incubated overnight in primary antibodies at 1:1,000 dilution in blocking buffer then washed three times for 10 min each in PBS 0.1% Tween and incubated for 1 h at room temperature in secondary antibody (GE Healthcare, Stockholm, Sweden). Finally, the PBS-Tween washes were repeated, and the membranes were developed with chemiluminescent ECL substrates. Antibodies used were as follows: CHD1 (#4351, Cell Signalling Technology, Danvers, MA, USA), CHD2 (#4170 Cell Signalling, Danvers, MA, USA), and H3 (ab1791, Abcam, Cambridge U.K.), all at a concentration of 1:1,000.

### DNase accessibility

To measure chromatin accessibility, we used the EpiQ™ chromatin analysis kit (BIORAD, Stockholm, Sweden) according to the manufacturer’s instructions at 28 hours post transfection with siRNA. Briefly, 0.25 × 10^6^ K562 cells were used per reaction and the resultant DNA was diluted to 1 in 400 in qPCR reactions. Accessibility was calculated as; 2^(Ct(undigested)-Ct(digested))^. We then normalized each loci to the percentage of accessibility in control siRNA treated cells. Primers were designed to DNase hypersensitivity peaks in K562 cells based on ENCODE data. For TSS, DHS regions we focused on expressed genes, whereas for intragenic and intergenic regions, we selected DHS sites observed when looking for accessible and inaccessible TSS regions. For tRNA loci, we chose loci from expressed tRNA genes that did not overlap with an annotated TSS but did overlap with a DHS. Primers are listed in Additional file 7.

### ChIP qPCR

K562 cells were fixed in 1% paraformaldehyde for 8 minutes, and ChIP was performed using the iDeal ChIP-seq kit (Diagenode, Liege, Belgium) according to the manufacturer’s instructions. Chromatin extracts from one million cells were sonicated to yield 100 to 500 bp fragments using a Bioruptor™ Plus (Diagenode) on high power for two rounds of 12 cycles with 30s on 30s off at 4°C. Antibodies used in ChIP were as follows; CHD1 - 3 μl per reaction of #4351 (Cell Signalling Technology, Danvers, MA, USA), CHD2 - 3 μl per reaction of ab68301 (Abcam, Cambridge U.K.), H3 - 2 μl per reaction of ab1791 (Abcam, Cambridge U.K.), and H3.3 - 3 μl per reaction of 09–838 (Millipore, Solna, Sweden). Primers for qPCR were designed across DNase hypersensitivity sites at TSS regions, intergenic or intragenic regions, or active tRNA loci and are listed in Additional file 7: Table S2.

## Electronic supplementary material

Additional file 1: **CHD7 occupancy at promoter chromatin states in K562 and H1 embryonic stem cells (ESCs).** The occupancy of CHD7 was calculated as the fold enrichment over input. (PDF 1 MB)

Additional file 2: **Recruitment of CHD1 and CHD2 to active tRNA loci.** (A) The occupancy of CHD1 and CHD2 across BRF1 and BRF2 binding sites in K562 cells was calculated as the fold enrichment over input. (B) A selected, expressed tRNA gene in an intergenic region showing BRF1, CHD1, CHD2 and Pol II occupancy in K562 cells. (PDF 1 MB)

Additional file 3: **Average enrichment of Pol II, Pol II ser2, CHD1 and CHD2 at transcribed genes.** Transcribed genes were identified as in the top quintile of Pol II ser 2 occupancy and analyzed for Pol II, CHD1 and CHD2 enrichment across the gene plus 2.5 kb up and downstream. (PDF 361 KB)

Additional file 4: **CHD1 and CHD2 siRNA knockdown.** (A) Transcript abundance of CHD1 and CHD2 relative to GAPDH in K562 cells 48 h following siRNA transfection. (B) Western blot analysis of CHD1 and CHD2 expression 72 h following transfection. (PDF 2 MB)

Additional file 5: **Analysis of H3.3 antibody specificity by western blot and ChIP-qPCR.** (A) The reactivity of total H3 and H3.3 specific antibodies toward 10 to 250 ng of recombinant human H3.1 and H3.3 was analyzed by western blot. (B) The relative enrichment of H3.3 to total H3 was examined by ChIP qPCR at active GAPDH and inactive MYOD gene TSS and the active NPM1 promoter and NPM1 intron. (PDF 1 MB)

Additional file 6: **ENCODE data files used.** (PDF 37 KB)

Additional file 7: **Primers for qPCR.** (PDF 40 KB)

## Abbreviations

CGI: CpG island
CHD: chromodomain helicase DNA binding protein
ChIP-seq: chromatin immunoprecipitation high throughput sequencing
DNase: deoxyribonucleic acid I
DHS: DNase hypersensitive site
DRB: 5,6-dichloro-1-β-D-ribofuranosyl-1H-benzimidazole
ENCODE: Encyclopedia of DNA Elements
ESC: embryonic stem cell
MACS: model-based analysis for ChIP-seq
MNase: micrococcal nuclease
NDR: nucleosome-depleted region
Pol II: RNA polymerase II
TSS: transcription start site.
