# Supplementary figures and images for: Transcription-coupled recruitment of human CHD1 and CHD2 influences chromatin accessibility and histone H3 and H3.3 occupancy at active chromatin regions
Source: Epigenetics Chromatin. 2015 Jan 15;8:4. doi: 10.1186/1756-8935-8-4 (PMC4305392; doi:10.1186/1756-8935-8-4)

K562

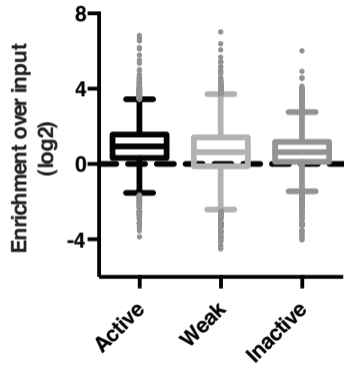

H1 ESCs

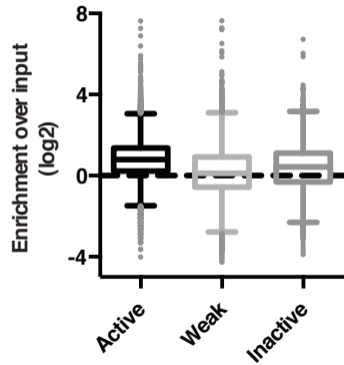

Supplement: Supplementary file 1 — Additional file 1: CHD7 occupancy at promoter chromatin states in K562 and H1 embryonic stem cells (ESCs). The occupancy of CHD7 was calculated as the fold enrichment over input. (PDF 1 MB) [file 13072_2014_346_MOESM1_ESM.pdf]

A

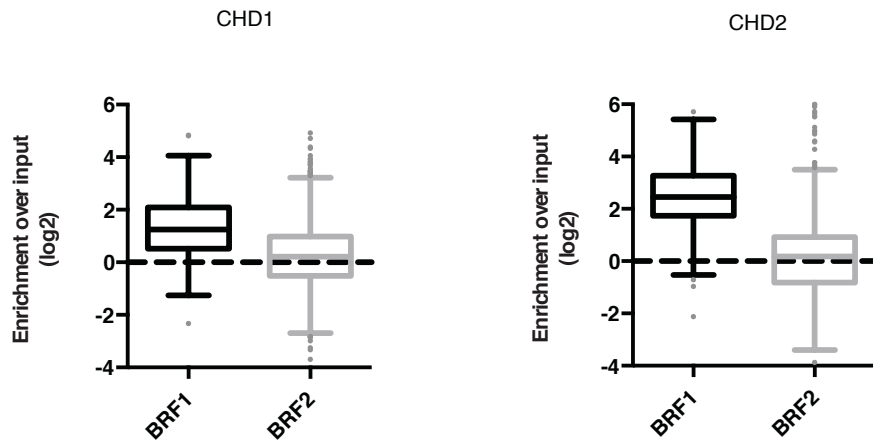

B

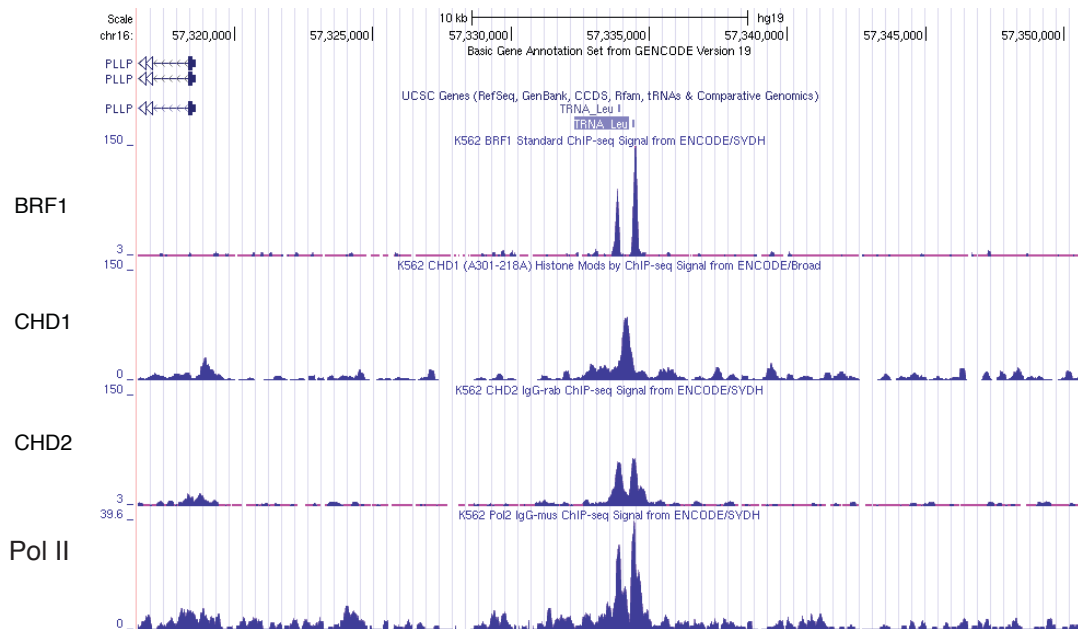

Supplement: Supplementary file 2 — Additional file 2: Recruitment of CHD1 and CHD2 to active tRNA loci. (A) The occupancy of CHD1 and CHD2 across BRF1 and BRF2 binding sites in K562 cells was calculated as the fold enrichment over input. (B) A selected, expressed tRNA gene in an intergenic region showing BRF1, CHD1, CHD2 and Pol II occupancy in K562 cells. (PDF 1 MB) [file 13072_2014_346_MOESM2_ESM.pdf]

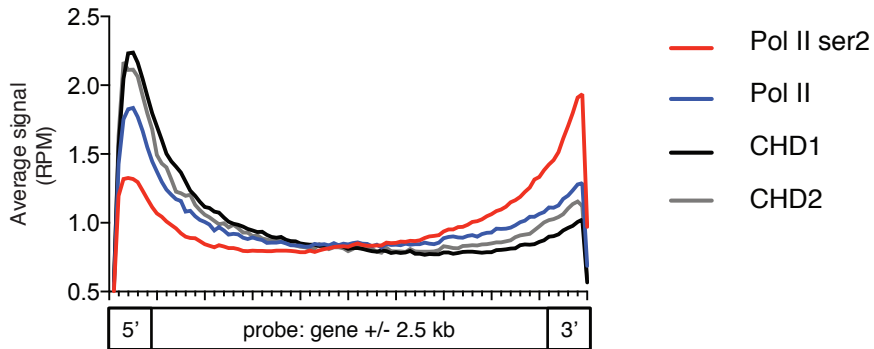

Supplement: Supplementary file 3 — Additional file 3: Average enrichment of Pol II, Pol II ser2, CHD1 and CHD2 at transcribed genes. Transcribed genes were identified as in the top quintile of Pol II ser 2 occupancy and analyzed for Pol II, CHD1 and CHD2 enrichment across the gene plus 2.5 kb up and downstream. (PDF 361 KB) [file 13072_2014_346_MOESM3_ESM.pdf]

A

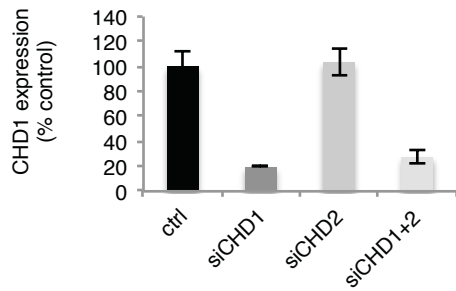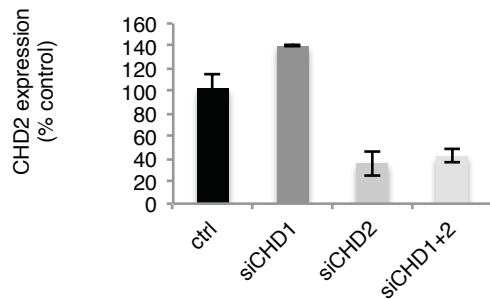

B

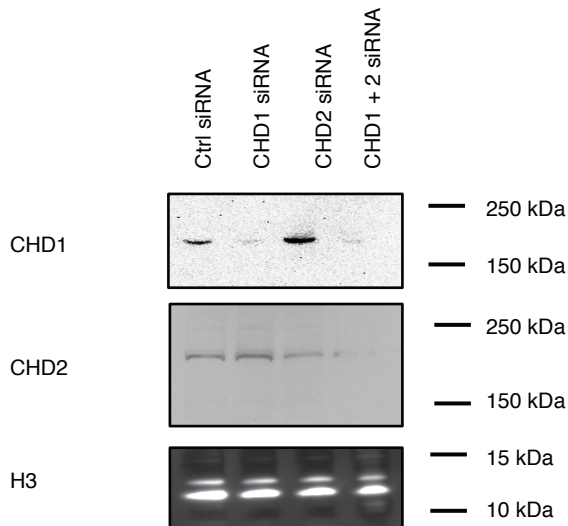

Supplement: Supplementary file 4 — Additional file 4: CHD1 and CHD2 siRNA knockdown. (A) Transcript abundance of CHD1 and CHD2 relative to GAPDH in K562 cells 48 h following siRNA transfection. (B) Western blot analysis of CHD1 and CHD2 expression 72 h following transfection. (PDF 2 MB) [file 13072_2014_346_MOESM4_ESM.pdf]

A

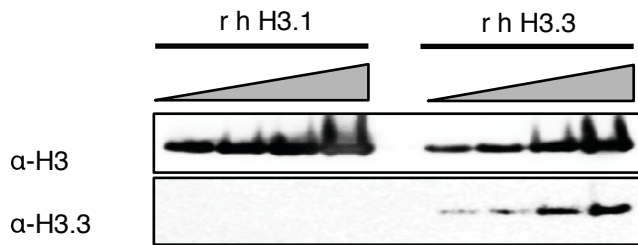

B

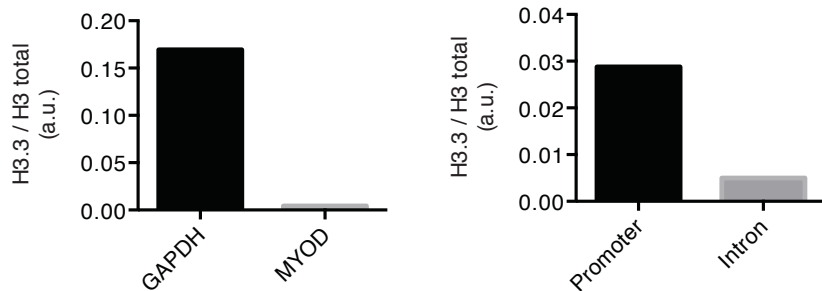

Supplement: Supplementary file 5 — Additional file 5: Analysis of H3.3 antibody specificity by western blot and ChIP-qPCR. (A) The reactivity of total H3 and H3.3 specific antibodies toward 10 to 250 ng of recombinant human H3.1 and H3.3 was analyzed by western blot. (B) The relative enrichment of H3.3 to total H3 was examined by ChIP qPCR at active GAPDH and inactive MYOD gene TSS and the active NPM1 promoter and NPM1 intron. (PDF 1 MB) [file 13072_2014_346_MOESM5_ESM.pdf]
