# Supplementary material for: Transcription-coupled recruitment of human CHD1 and CHD2 influences chromatin accessibility and histone H3 and H3.3 occupancy at active chromatin regions
Source: Epigenetics Chromatin. 2015 Jan 15;8:4. doi: 10.1186/1756-8935-8-4 (PMC4305392; doi:10.1186/1756-8935-8-4)
Supplement: Supplementary file 6 — Additional file 6: ENCODE data files used. (PDF 37 KB) [file 13072_2014_346_MOESM6_ESM.pdf]

## Supplementary table 1 ENCODE data files used

| Data | ENCODE Project File |
|---|---|
| | |
| **K562 cells** | |
| | |
| Chromatin state | wgEncodeBroadHmmK562HMM.bed |
| RNA-seq | wgEncodeCaltechRnaSeqK562R1x75dAlignsRep1V2.bam |
| Short RNA-seq | K562_WholeCell_Short_RNA_CSHL_Contig.CSHL_LID10567-001WC-r1.LID10568-002WC-r2.idrFilt0.1.bb |
| Input | wgEncodeBroadHistoneK562ControlStdAlnRep1.bam |
| Input | wgEncodeSydhTfbsK562InputIggmusAln.bam |
| Input | wgEncodeSydhTfbsK562InputIggrabAln.bam |
| H3K4me1 | wgEncodeBroadHistoneK562H3k4me1StdAlnRep1.bam |
| H3K4me2 | wgEncodeBroadHistoneK562H3k4me2StdAlnRep1.bam |
| H3K4me3 | wgEncodeBroadHistoneK562H3k4me3StdAlnRep1.bam |
| CHD1 | wgEncodeBroadHistoneK562Chd1a301218aStdAlnRep1.bam |
| CHD2 | wgEncodeSydhTfbsK562Chd2ab68301IggrabAlnRep1.bam |
| Pol II | wgEncodeSydhTfbsK562Pol2IggmusAlnRep1.bam |
| BRF1 | wgEncodeSydhTfbsK562Brf1StdAlnRep1.bam |
| BRF2 | wgEncodeSydhTfbsK562Brf2StdAlnRep1.bam |
| DNase-seq | wgEncodeOpenChromDnaseK562AlnRep1V2.bam |
| MNase-seq | wgEncodeSydhNsomeK562AlnRep1.bam |
| | |
| **H1 ESCs** | |
| | |
| Chromatin state | wgEncodeBroadHmmH1hescHMM.bed |
| Input | wgEncodeBroadHistoneH1hescControlStdAlnRep1.bam |
| Input | wgEncodeSydhTfbsH1hescInputIggrabAln.bam |
| CHD1 | wgEncodeBroadHistoneH1hescChd1a301218aStdAlnRep2.bam |
| CHD2 | wgEncodeSydhTfbsH1hescChd2IggrabAlnRep1.bam |
| | |
| **GM12878** | |
| | |
| Chromatin state | wgEncodeBroadHmmGm12878HMM.bed |
| Input | wgEncodeBroadHistoneGm12878ControlStdAlnRep1.bam |
| Input | wgEncodeSydhTfbsGm12878InputIggmusAlnRep1.bam |
| CHD1 | wgEncodeSydhTfbsGm12878Chd1a301218aIggmusAlnRep1.bam |
| CHD2 | wgEncodeSydhTfbsGm12878Chd2ab68301IggmusAlnRep1.bam |
| | |
