# Supplementary material for: Transcription-coupled recruitment of human CHD1 and CHD2 influences chromatin accessibility and histone H3 and H3.3 occupancy at active chromatin regions
Source: Epigenetics Chromatin. 2015 Jan 15;8:4. doi: 10.1186/1756-8935-8-4 (PMC4305392; doi:10.1186/1756-8935-8-4)
Supplement: Supplementary file 7 — Additional file 7: Primers for qPCR. (PDF 40 KB) [file 13072_2014_346_MOESM7_ESM.pdf]

Supplementary table 2 qPCR primers used in DNase-qPCR and H3 and H3.3 ChIP-qPCR

Region	Forward	Reverse
**TSS DHS sites**		
C1ORF159	GAAGCGCCCTGGGAAATGTA	GGGAATGTAGTCCCTCCGTG
EGR1	CTGCTCAGTTCGTGCTCACT	CTCCTCGCTCCTCCTCCC
NPM1	GCGAGGTAGAAAGGAGTGGG	GACGGAATCACACCAGGGAA
GCAT	ACAAGTCAAGCCTCTCGGTC	TCGCTTAAAAAGGCGGGGAG
NRAS	CCCGCTACGTAATCAGTCGG	CATGACTCGTGGTTCGGAGG
HGB1	TTCTTCATCCCTAGCCAGCC	CCTTGCCTTGACCAATAGCC
PIN1	AGGGATAACGTGGGAGGGTC	CTAACGGTCCGGATTGGCTT
HES4	CCTCAGGCCGTTTCCCTATT	GCGAGTGTGGGAAAGAATGC
H3F3B	TGACATCCCCCGACACAAAG	GCCAATCAGCGGCGTAGATA
OGG1	CGCCCACCCTGATTTCTCAT	AACCACCGCTCATTTCACCT
HGB2	GTGTGGAACTGCTGAAGGGT	GCCTTGCCTTGACCAATAGC
NCL	TGAAGATCCCGGAGCACGTA	TTCCACAGGCGATTACTGGG
KIAA0895L	AAATGAGAGGGTGTGGCCTG	TTCCCCATTGGCTCTTCACC
DNMT3B	CCGAGCGATTTCAAATTTCCCT	GAGTGGGTGGGGAGGGG
BZRAPP	AATAGGAAGTCGGCTGCTGG	CTGCTCTTCCTTCTGTCCCC
**Intra and Intergenic**		
Intragenic 1	TTTGCATGGGGCTCATTTGC	GGATTGACAGTGGCTCCTCC
Intragenic 2	CGTGCGGTGAGCGGG	CAGCCTCGCTCCATCTCATA
Intragenic 3	GTCAGGTGGTCAGCTTCTCC	CTGGCTCAAGCACAGCAATG
Intragenic 4	CAGGACAGGACGGGGAATTT	AAGTGGCGGTGATTACCCTG
Intragenic 5	CAGAACACTCACAGGCAACG	GAAGGGGGAAGGATCCAAGC
Intragenic 6	TGGAAGGGAACTCCGAAGGA	CCATCTCTCCCGGTCGAACA
Intergenic 1	CTGCTGCCGCATTTTCCTTT	AGATAAGGCCTCTGGCTGGA
Intergenic 2	CCACTCTCCTCATCCCTCCA	TACGACTTCAGGCTCTGGGT
Intergenic 3	TGGAAACGGGTGTCTGCTAC	CCAGCGGTCTTTTCCACCTC
Intergenic 4	TCAGCGGAGAGTACCTGGTT	GTTTAGGGATGCTGCCACCT
Intergenic 5	GGTCGGCCTCGGGTCT	GGGCCGCAGAAACACCA
Intergenic 6	ATGCCTGCGAGGATGGAAAA	ACTCTGCTCACCAAACCTGG
Intergenic 7	CTCCCACTGCCTTCTGTTGT	CTGTGAGCACGGACATAGCC
Intergenic 8	AGCTTATTCAGTGCCTGGGG	GGGAAAGGTCAGATGCGAGG
Intergenic 9	GTCCTGACCTGGCCACC	GTCGAGGAAGTGGCGTTTTC
**Active tRNA**		
tRNA 1	ACACCCCACCACTATCTCCA	TTCTTCCCAGGTGCCTCAAC
tRNA 2	CCCACCAATATCTCCACCCAC	CCAGATGCCAATGATTGCGG
tRNA 3	ACTCCAGTTTCTTGCCCAGG	GCCACGACTACACGAATGGA
tRNA 4	CGTTGCCAGTGAAGGAGGAT	GCCCCTCTCTAATTCTGCCC
tRNA 5	TAGACGGAAACCAGTGCGTC	CCTTCATTCCTCGGACGCTT
tRNA 6	CGGGTGAAGGAAAACTGGGA	CAAACGGCATGCTTTGTGGA
